# Therapeutic Implications of Targeting Heat Shock Protein 70 by Immunization or Antibodies in Experimental Skin Inflammation

**DOI:** 10.3389/fimmu.2021.614320

**Published:** 2021-02-23

**Authors:** Stefan Tukaj, Jagoda Mantej, Michał Sobala, Katarzyna Potrykus, Zbigniew Tukaj, Detlef Zillikens, Ralf J. Ludwig, Katja Bieber, Michael Kasperkiewicz

**Affiliations:** ^1^ Department of Molecular Biology, Faculty of Biology, University of Gdańsk, Gdańsk, Poland; ^2^ Department of Bacterial Molecular Genetics, Faculty of Biology, University of Gdańsk, Gdańsk, Poland; ^3^ Department of Plant Physiology and Biotechnology, Faculty of Biology, University of Gdańsk, Gdańsk, Poland; ^4^ Department of Dermatology and Center for Research on Inflammation of the Skin, University of Lübeck, Lübeck, Germany; ^5^ Lübeck Institute of Experimental Dermatology and Center for Research on Inflammation of the Skin, University of Lübeck, Lübeck, Germany; ^6^ Department of Dermatology, Keck School of Medicine, University of Southern California, Los Angeles, CA, United States

**Keywords:** psoriasis, regulatory T cells, Treg, Th17, heat shock proteins, Hsp70, immunization

## Abstract

Heat shock proteins (Hsp) are constitutive and stress-induced molecules which have been reported to impact innate and adaptive immune responses. Here, we evaluated the role of Hsp70 as a treatment target in the imiquimod-induced, psoriasis-like skin inflammation mouse model and related *in vitro* assays. We found that immunization of mice with Hsp70 resulted in decreased clinical and histological disease severity associated with expansion of T cells in favor of regulatory subtypes (CD4^+^FoxP3^+^/CD4^+^CD25^+^ cells). Similarly, anti-Hsp70 antibody treatment led to lowered disease activity associated with down-regulation of pro-inflammatory Th17 cells. A direct stimulating action of Hsp70 on regulatory T cells and its anti-proliferative effects on keratinocytes were confirmed in cell culture experiments. Our observations suggest that Hsp70 may be a promising therapeutic target in psoriasis and potentially other autoimmune dermatoses.

## Introduction

Heat shock proteins (Hsp) are a diverse group of constitutive and stress-induced molecules that are categorized into several families named on the basis of their molecular weight, including Hsp110, Hsp90, Hsp70, Hsp60, Hsp40, and the so-called small Hsp. Hsp act as intracellular chaperones being involved in protein folding and homeostasis but can also be released to extracellular compartments upon stressful conditions or cell death (1).

Given their additional complex immunological roles both inside and outside of cells, multiple studies particularly identified Hsp90 and Hsp70 as important pathophysiological factors and treatment targets of different chronic inflammatory and autoimmune disorders (2–4). Using models of the prototypical autoimmune blistering disease epidermolysis bullosa acquisita, we have previously comprehensively shown that pharmacological Hsp90 inhibition results in attenuation of disease activity by multimodal anti-inflammatory mechanisms (2). It is assumed that the immunomodulatory effects of Hsp90 inhibition are at least partly mediated by the up-regulation of intracellular Hsp70 (a surrogate marker of Hsp90 blockade) which inhibits the nuclear factor-κB (NF-κB) inflammatory pathway (3). In addition, increased Hsp70 expression achieved independently of Hsp90 inhibition (e.g., by Hsp70 vaccination) has been demonstrated to be associated with down-regulation of inflammatory processes in several preclinical models of autoimmune diseases (4–6). However, some contradictory results suggested that both intra- and extracellular Hsp70 can exert a dual role in autoimmune diseases (i.e., either promoting or silencing immune responses), depending on its origin (i.e., bacterial or self), site of inflammation, type of disease, and possibly other undefined reasons (4).

Evidence suggests involvement of Hsp, including Hsp70, in the development of psoriasis (7), an autoimmune-associated chronic inflammatory skin disease characterized by impaired immunological cell function with altered Th17/regulatory T cell (Treg) balance, autoreactive T cells, and dysregulation of keratinocyte proliferation (8). In this disease, increased expressions of Hsp and immune responses to these proteins have been described (7). Recently, an equivocal role of Hsp70 has been demonstrated in the imiquimod (IMQ)-induced skin inflammation mouse model which has become the most widely used murine model for preclinical studies of psoriasis-like dermatitis (9–11). While one study showed that topical application of Hsp70 led to a reduction of skin lesions and inflammatory markers (10), another study described similar effects using a topical Hsp70 inhibitor (9). Here, we further defined the role of Hsp70 (using murine [m]-, human [h]-, or plant [p]-Hsp70) as a treatment target in the IMQ mouse model and related *in vitro* assays.

## Materials and Methods

### Cloning, Expression, and Purification of Hsp70

Full-length synthetic DNA fragments encoding Hsp70 from *Nicotiana tabacum* (BAM24707.1), *Mus musculus* (NP_034609.2), and *Homo sapiens* (NP_005336.3) have been obtained from Thermo Scientific (GeneArt service). Codon usage was optimized for efficient gene expression in *E. coli* by the GeneOptimizer software. The inserts were synthesized with N-terminal 6x-His-SUMO tag and cloned into the pET151/D-TOPO or pRSET_A_A185 (Thermo Scientific) plasmid. Genetically modified lipopolysaccharide-free *E. coli* ClearColi^®^ BL21(DE3) (Lucigen) strain carrying the respective plasmid was grown in the LB medium supplemented with 1% NaCl, 1 mM IPTG (Sigma), and ampicillin at 18°C overnight. Cells were harvested by centrifugation, resuspended in a lysis buffer, and disrupted by sonication. After centrifugation, the supernatant was loaded on the HIS-Select^®^ Nickel Affinity Gel resin (Sigma) equilibrated with the lysis buffer. To remove unbound proteins and the chaperone-associated substrates, the column was washed with a buffer containing 5 mM ATP, 5 mM MgCl_2_, 1 M NaCl, and 20 mM Tris-HCl pH=8.0. The Hsp70 containing fractions (eluted with lysis buffer containing 180 mM imidazole) were dialyzed against a dialysis buffer (20 mM Tris-HCl pH=8.0, 250 mM NaCl, 10% glycerol), followed by His-tag cleavage using SUMO protease (Sigma). To remove His-tag from the mixture, the protein sample was loaded on the HIS-Select^®^ Nickel Affinity Gel resin (Sigma) equilibrated with the dialysis buffer. The Hsp70 fraction (99% purity) was filtered (0.22 μm) and stored at −80°C for further analysis. In addition, Hsp70 from *Nicotiana tabacum* leaves has been purified using ATP-agarose, as described previously (12).

### Flow Cytometric Immunophenotyping

Single-cell suspensions from spleen of mice were stained with anti-CD4 (clone GK1.5; BioLegend), anti-CD25 (clone 3C7; BioLegend), and anti-FoxP3 (clone MF-14; BioLegend). In the case of intracellular cytokine staining, splenocytes were cultured in RPMI 1640 medium containing 10% fetal calf serum, 2 mM L-glutamine, 100 U/ml penicillin, and 100 U/ml streptomycin in presence of phorbol-12-myristate-13-acetate (PMA) (50 ng/ml; Sigma), ionomycin (1 μg/ml; Sigma), and monensin (BioLegend) for 5 h. Cells were washed, fixed, permeabilized, and stained with anti-IL-17 (clone TC11-18H10.1; BioLegend). Viable single cells were analyzed based on forward and side light scatter properties with a CyFlow Cube 6 flow cytometer (Sysmex) or MACSQuant Analyzer 10 flow cytometer (Miltenyi Biotec).

### Detection of Circulating Anti-Hsp70 IgG

Serum levels of IgG against Hsp70 were evaluated by home-made enzyme-linked immunosorbent assay (ELISA), as described previously with minor modifications (13). Briefly, medium-binding 96-well plates were coated with m-Hsp70 at a concentration of 0.5 μg/ml in 0.1 M bicarbonate buffer at 4°C for 18 h. The wells were blocked with 1% bovine serum albumin (BSA) in phosphate-buffered saline (PBS) at room temperature (RT) for 2 h. After being washed with PBS + 0.05% Tween 20, mouse sera diluted 1:100 in PBS + 0.1% BSA were incubated at RT for 1 h. Plates were then incubated with horseradish peroxidase (HRP)-conjugated anti-mouse IgG (Sigma) antibodies diluted 1:5000 in PBS containing 0.1% BSA at RT for 1 h. TMB substrate solution (Sigma) was used to visualize HRP enzymatic reaction, and the reaction was stopped by 0.5 M H_2_SO_4_. Optical density measurements were performed at 450 nm with an ELISA plate reader.

### Mice

Female BALB/c mice aged 6 weeks were purchased from the Tri-City Academic Laboratory Animal Centre - Research and Services Centre (Gdańsk, Poland). Animal experiments were approved by local authorities of the Animal Care and Use Committee (Bydgoszcz, Poland) and performed by certified personnel in the animal facility of the University of Gdańsk, Poland.

### Disease Induction and Treatment

To induce psoriasis-like skin inflammation, a 2 x 3 cm area on the mouse back was shaved and depilated on day −2 of the experiment. Starting on day 0, 50 mg Aldara™ cream, containing 5% IMQ (Meda AB, Sweden) was topically applied to the shaved back skin daily for six consecutive days. Skin inflammation was evaluated daily using a modified version of the Psoriasis Activity and Severity Index (PASI), as described previously (14). Briefly, erythema, infiltration, and desquamation were each scored independently by two blinded persons on a scale from 0 to 4: 0, none; 1, minimal; 2, mild; 3, distinct; and 4 severe. The scores of these individual aspects of dermatitis were summed up to calculate the cumulative score from 0 to 12.

Fourteen days prior to the first Aldara™ application, mice were treated with a single subcutaneous injection of 100 μg recombinant m-Hsp70 or p-Hsp70, PBS, or 100 μg control ovalbumin (OVA) (Sigma) emulsified in 2 mg of adjuvant dimethyl dioctadecyl ammonium bromide (DDA) (Sigma). The use of ClearColi cells warrants that the purified overproduced Hsp70 is free of lipopolysaccharide contaminants.

In a functional assay, one day prior to the 6-day Aldara™ treatment, naive mice were treated with a single intraperitoneal injection of 50 μg mouse anti-Hsp70 IgG_1_ mAb (clone BRM-22; Sigma) or 50 μg IgG_1_ isotype control (Sigma) in PBS.

### Histopathology

For histopathology, skin samples of the back obtained on the final day of the experiments were fixed in 4% (w/v) buffered formalin and embedded in paraffin. 6-μm tissues sections were stained with hematoxylin and eosin (H&E). Dermal leukocyte infiltration and epidermal thickness were scored blindly by an independent researcher on a scale from 0 to 4: 0, none; 1, slight; 2, moderate; 3, marked; and 4, very marked, as described previously (14).

### Cell Culture

Splenocytes of naive BALB/c mice were cultured in RPMI 1640 medium containing 10% fetal calf serum, 2 mM L-glutamine, 100 U/ml penicillin, and 100 U/ml streptomycin in the presence of 1 μg/ml immobilized anti-CD3 mAb and 1 μg/ml soluble anti-CD28 mAb in 24-well culture plates at 5% CO_2_ and 37°C without (control) or with 20 μg/ml of substrate- and endotoxin-free, non-recombinant *N. tabacum*-derived Hsp70 for 72 h.

HaCaT cells were cultured in DMEM medium (Sigma) at 37°C in 5% CO_2_ atmosphere. Cells were seeded on 96‐well plates and grown to 80% confluence. Cells were incubated with IMQ (50 µM; Sigma) and cultured in the absence or presence of different concentrations of recombinant h-Hsp70 or p-Hsp70.

### Proliferation Assay

Cell proliferation was assayed by ELISA after BrdU (Roche) incorporation at 18 h of IMQ (Abcam) treatment, followed by 6 h of incubation according to manufacturer’s protocol.

### Cytokine Measurement

IL‐8 levels were analyzed in cell culture supernatants by ELISA (BioLegend).

### Statistical Analysis

Statistical analyses were performed using GraphPad Prism 5 (San Diego, CA). The Shapiro-Wilk test was used to verify whether the data had normal distribution. Normal and non-normal distributed data was analyzed by Student’s t-test and Mann Whitney U test or Kruskal Wallis test, respectively. P values less than 0.05 were considered statistically significant.

## Results

### Hsp70 Immunization Results in Decreased Disease Severity Associated With Expansion of T Cells in Favor of Regulatory Subtypes in the Imiquimod Mouse Model

**Figure 1:**
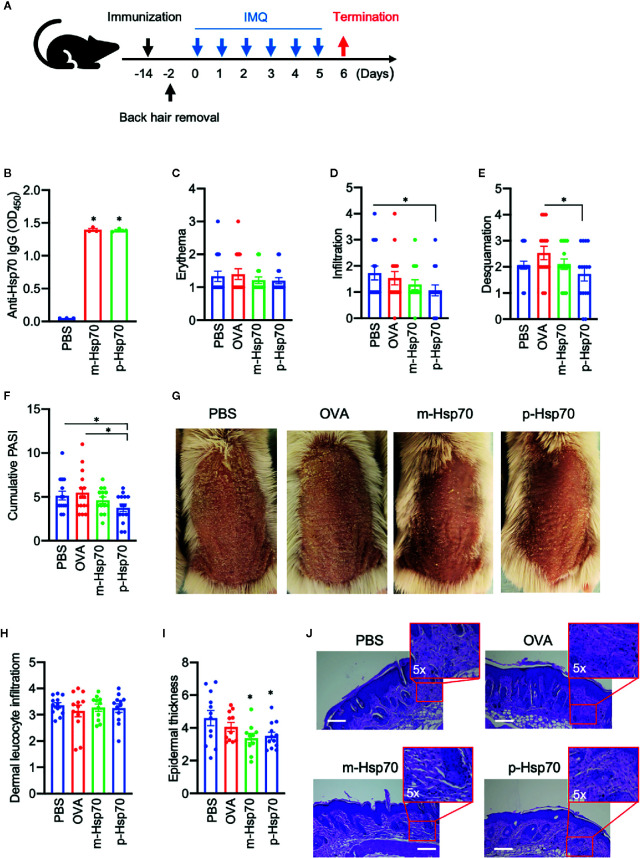
Hsp70 immunization attenuates experimental psoriasis-like dermatitis. **(A)** Schematic illustration of the experimental setup. Mice were treated with a single subcutaneous injection of murine [m]-Hsp70, plant [p]-Hsp70, phosphate-buffered saline (PBS), or ovalbumin (OVA), followed (after two weeks) by daily application of topical imiquimod (IMQ) on shaved backs for 6 consecutive days. **(B)** Anti-Hsp70 IgG serum levels fourteen days after immunization of mice with Hsp70 or PBS injection of controls, as analyzed by enzyme-linked immunosorbent assay (ELISA). Clinical disease severity of PBS-, OVA-, and Hsp70-treated mice shown by scores for **(C)** erythema, **(D)** infiltration, **(E)** and desquamation, as well as **(F)** the resulting cumulative PASI score at the of the experiment. **(G)** Representative clinical presentations. Histological disease severity shown by scores for **(H)** dermal leukocyte infiltration and **(I)** epidermal thickness at the end of the experiment. **(J)** Representative hematoxylin and eosin (H&E) staining of skin biopsies with corresponding higher magnifications of the dermis. Data are representative of three independent experiments and expressed as mean ± SEM of a total of 10–15 mice per group. Dot plots overlaid on bar graphs represent individual data points. *P < 0.05. Bars = 100 μm.

**Figure 2:**
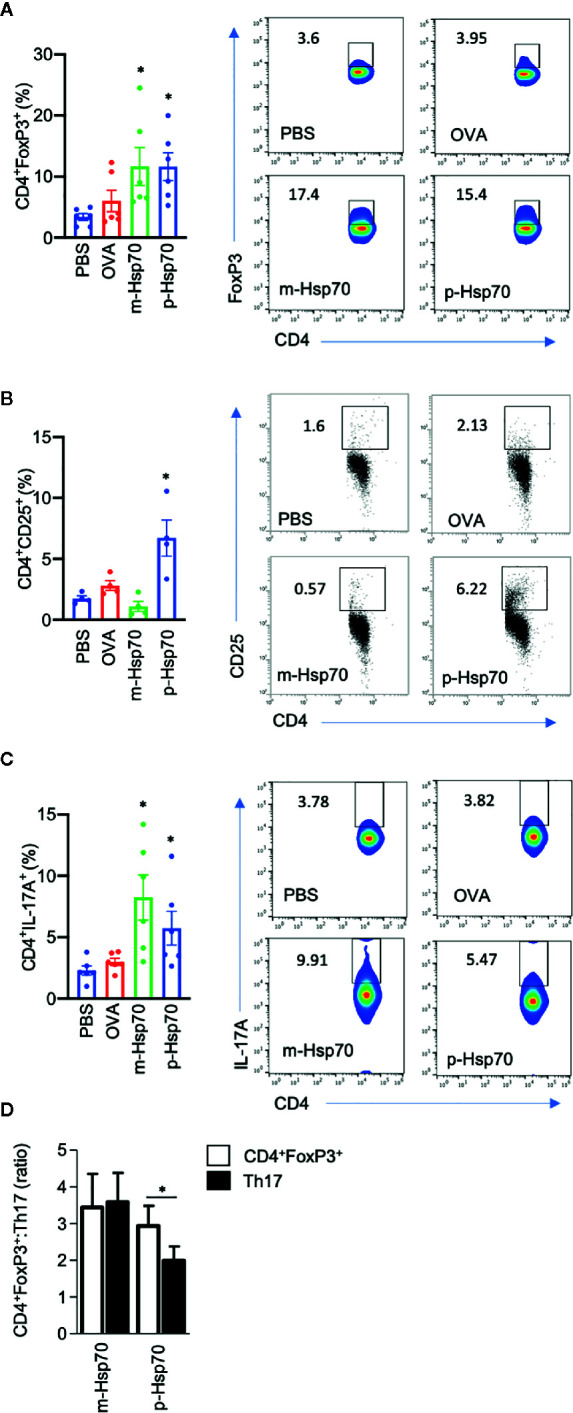
Hsp70 immunization is associated with a predominant increase in regulatory subtypes of T cells. Bar charts show percentages of **(A)** splenic CD4^+^FoxP3^+^ cells, **(B)** blood CD4^+^CD25^+^ cells, **(C)** splenic CD4^+^IL-17A^+^ cells, as well as **(D)** CD4^+^FoxP3^+^:Th17 ratio at the end of the imiquimod (IMQ)-induced skin inflammation mouse experiment, as analyzed by flow cytometry. The numbers in the gates of the representative results (right) are the percentages of the respective cell populations. To express the ratio, data were normalized to a mean value of the control group [phosphate-buffered saline (PBS)-treated mice]. Data are expressed as mean ± SEM of four to six mice per group. Dot plots overlaid on bar graphs represent individual data points. *P < 0.05.

The effects of immunization with a highly pure, substrate-, and endotoxin-free recombinant m-Hsp70 and p-Hsp70 were tested in the IMQ-induced skin inflammation mouse model (**Figure 1A**). Immunization of mice with either m-Hsp70 or p-Hsp70 led to the generation of circulating anti-Hsp70 IgG (**Figure 1B**). Clinical disease severity (i.e., infiltration, desquamation, and cumulative PASI) was significantly reduced in p-Hsp70- but not m-Hsp70-treated animals when compared with PBS- and OVA-injected animals (**Figures 1C–G**). Histologically, epidermal hyperplasia was significantly milder in both m-Hsp70- and p-Hsp70-treated mice compared with PBS- and OVA-treated control animals, whereas dermal leukocyte infiltration was not altered by Hsp70 immunization (**Figures 1H–J**).We next investigated whether Hsp70 immunization had an impact on Tregs (i.e., splenic CD4^+^FoxP3^+^ cells and circulating CD4^+^CD25^+^ cells) and pro-inflammatory splenic CD4^+^IL-17^+^ T cells. Both m-Hsp70- and p-Hsp70-immunized mice had significantly increased frequencies of CD4^+^FoxP3^+^ and pro-inflammatory T cells when compared to PBS-treated control mice (**Figures 2A–C**). However, only immunization with p-Hsp70 was associated with a significant expansion of both types of Tregs and a significant increase of the CD4^+^FoxP3^+^:Th17 ratio (**Figures 2A–D**).

### Plant-Derived Hsp70 Induces CD4^+^CD25^+^ and Inhibits Th17 Cells *In Vitro*

**Figure 3:**
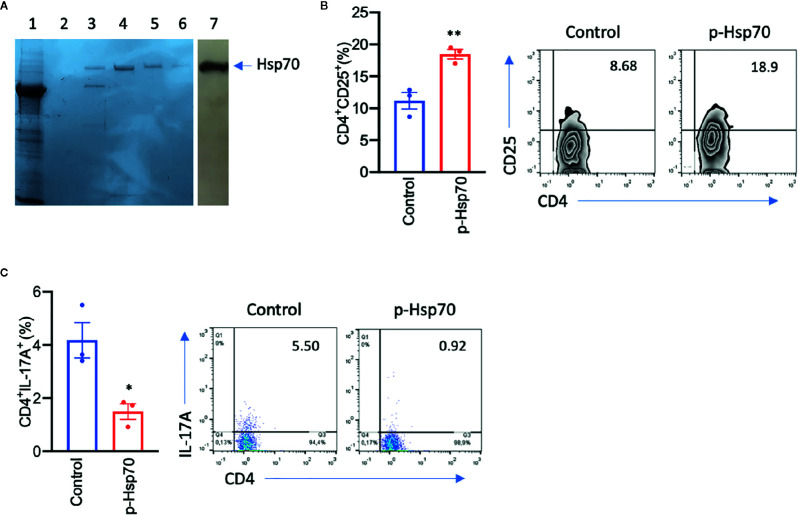
Plant [p]-Hsp70 induces CD4^+^CD25^+^ and inhibits Th17 cells *in vitro*. **(A)** Non-recombinant p-Hsp70 from *Nicotiana tabacum* leaves has been purified using ATP-agarose. Standard Coomassie Brilliant Blue R250 staining method was used, with lane no. 1 representing whole cell lysates, lanes no. 2–6 purification steps, and lane no. 7 immunodetection of [p]-Hsp70 using anti-Hsp70 antibodies. Bar charts show percentages of **(B)** CD4^+^CD25^+^ or **(C)** CD4^+^IL-17A^+^cells. The numbers in the gates of the representative results (right) are the percentages of the respective cell populations. Data are expressed as mean ± SEM of three mouse donors. Dot plots overlaid on bar graphs represent individual data points. *P < 0.05, **P < 0.01.

Since the above observations concerning p-Hsp70 are based on recombinant protein preparations, we investigated whether non-recombinant Hsp70 directly obtained from *Nicotiana tabacum* leaves had a similar immunomodulatory activity. We found that such Hsp70 preparation led to induction of CD4^+^CD25^+^ cells and reduced the frequency of the Th17 population in anti-CD3/CD28-stimulated spleen cultures (**Figures 3A–C**).

### Anti-Hsp70 Antibodies Lead to Lowered Disease Activity Associated With Down-Regulation of Pro-Inflammatory T cells in the Imiquimod Mouse Model

**Figure 4:**
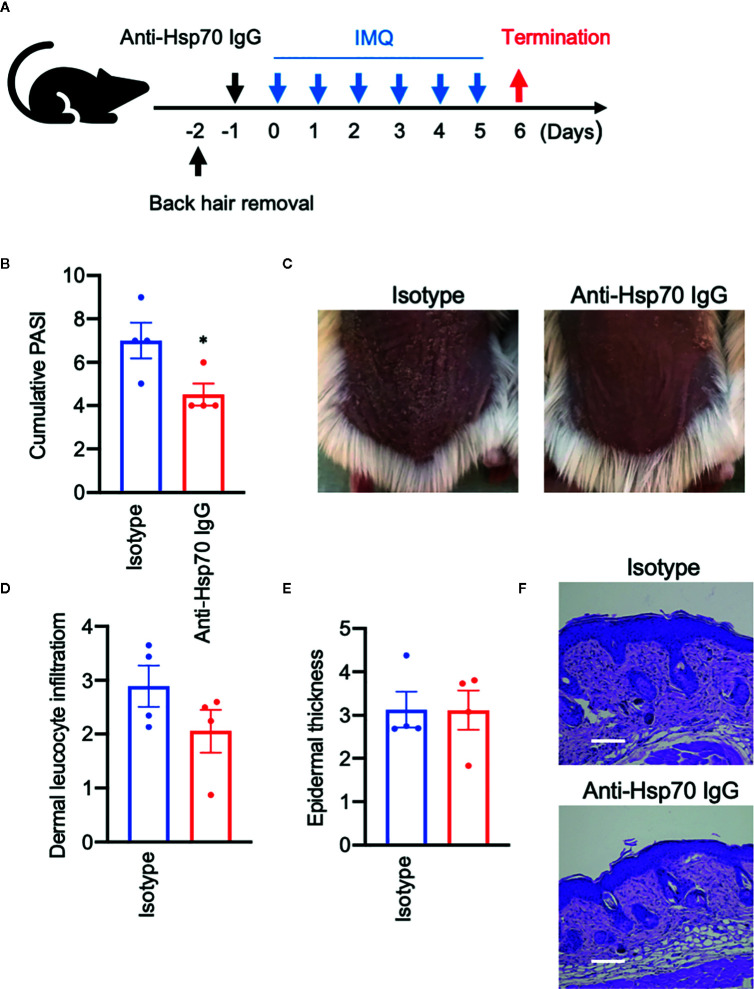
Anti-Hsp70 antibodies ameliorate experimental psoriasis-like dermatitis. **(A)** Schematic illustration of the experimental setup. In a functional assay, naive mice were treated with a single intraperitoneal injection of mouse anti-Hsp70 IgG_1_ mAb or isotype control (IgG_1_), followed (next day) by daily application of topical imiquimod (IMQ) on shaved backs for six consecutive days. **(B)** Clinical disease severity of isotype- and anti-Hsp70 IgG-treated mice shown by the cumulative PASI score at the end of the experiment. **(C)** Representative clinical presentations. Histological disease severity shown by scores for **(D)** dermal leukocyte infiltration and **(E)** epidermal thickness at the end of the experiment. **(F)** Representative hematoxylin and eosin (H&E) staining of skin biopsies. Data are expressed as mean ± SEM of one experiment using four mice per group. Dot plots overlaid on bar graphs represent individual data points. *P < 0.05. Bars = 100 μm.

To evaluate the role of anti-Hsp70 antibodies in the IMQ mouse model, that are significantly induced upon immunization of animals with Hsp70, naive mice were injected with murine anti-Hsp70 IgG or IgG isotype control one day prior to the IMQ treatment (**Figure 4A**). We found that anti-Hsp70 IgG-treated mice had a significantly lower PASI scores when compared to control mice (**Figures 4B, C** **)**. Histologically, there was a trend toward a significant (p=0.09) decrease in the dermal inflammatory cell infiltrate without influence on epidermal thickness in the anti-Hsp70 IgG-treated mice when compared to controls (**Figures 4D–F**).

**Figure 5:**
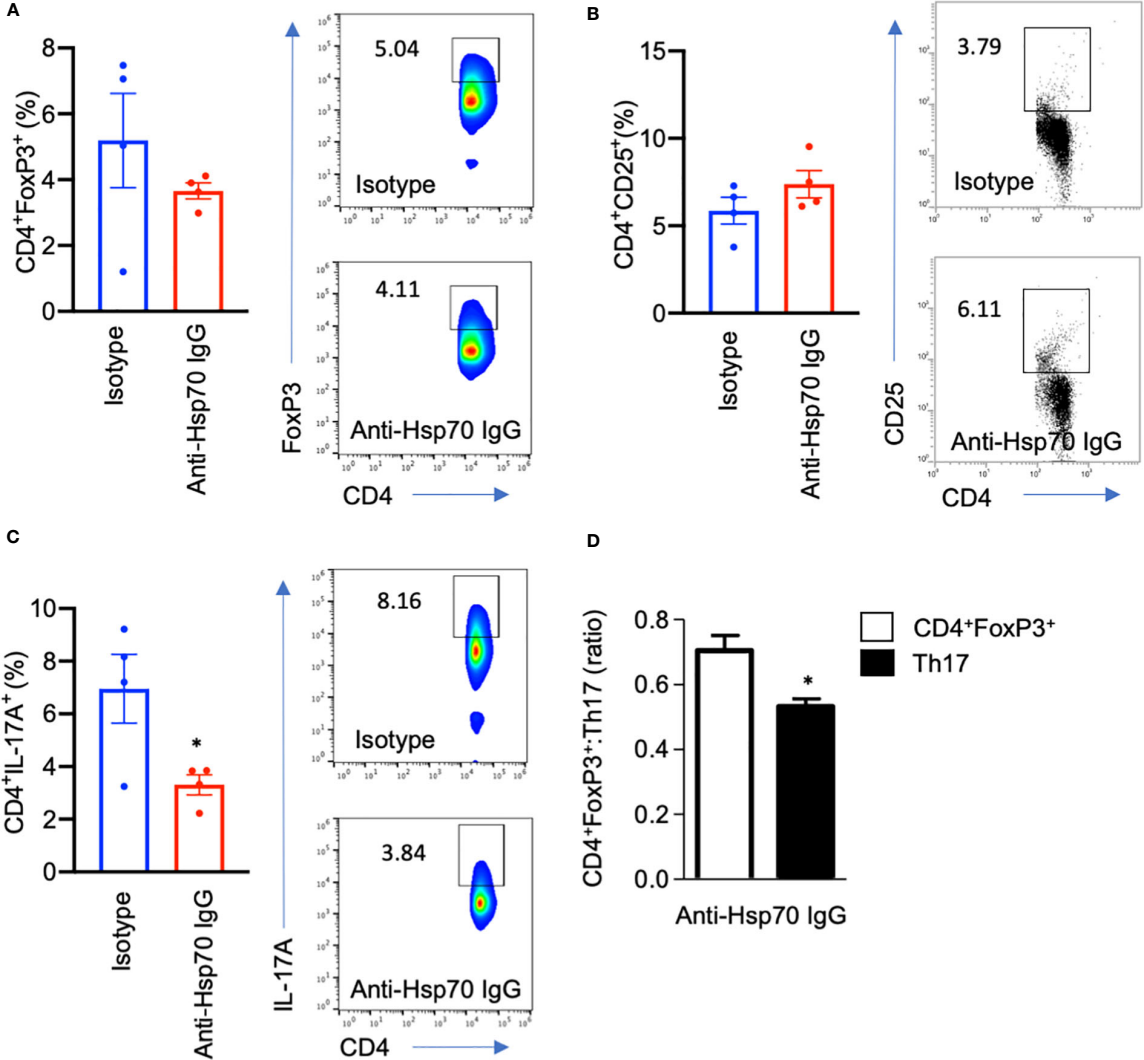
Anti-Hsp70 antibody treatment is associated with a decrease in pro-inflammatory T cells. Bar charts show percentages of **(A)** splenic CD4^+^FoxP3^+^ cells, **(B)** blood CD4^+^CD25^+^ cells, **(C)** splenic CD4^+^IL-17^+^ cells, as well as **(D)** CD4^+^FoxP3^+^:Th17 ratio at the end of the imiquimod (IMQ)-induced skin inflammation mouse experiment, as analyzed by flow cytometry. The numbers in the gates of the representative results (right) are the percentages of the respective cell populations. To express the ratio, data were normalized to a mean value of the control group (isotype-treated mice). Data are expressed as mean ± SEM of one experiment using four mice per group. Dot plots overlaid on bar graphs represent individual data points. *P < 0.05.

Immunophenotyping analysis revealed that anti-Hsp70 treatment had no significant effect on splenic CD4^+^FoxP3^+^ or blood CD4^+^CD25^+^ cell frequencies but was associated with a significantly lower percentage of splenic Th17 cells and a significant increase of the CD4^+^FoxP3^+^:Th17 ratio (**Figures 5A–D**).

### Hsp70 Reduces Proliferation and IL-8 Secretion of Imiquimod-Stimulated Human Keratinocyte (HaCaT) Cells

**Figure 6:**
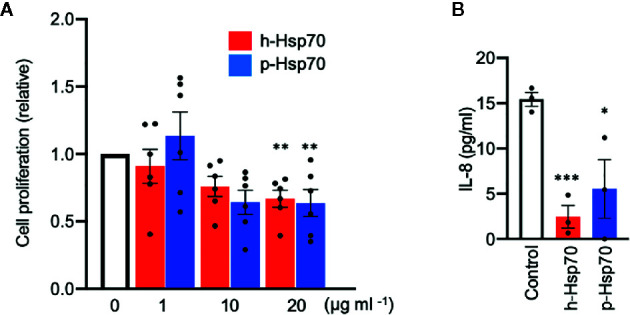
Hsp70 inhibits proliferation and IL-8 secretion of imiquimod (IMQ)-stimulated HaCaT cells *in vitro*. **(A)** Proliferation response of HaCaT cells stimulated by IMQ (50 µmol) in the absence and presence of different concentrations of human [h]-Hsp70 and plant [p]-Hsp70. Cell proliferation was assayed by enzyme-linked immunosorbent assay (ELISA) after BrdU incorporation at 18 h of IMQ treatment, followed by 6 h incubation. Results are mean ± SEM of two independent experiments, each performed in triplicate. **P < 0.01. **(B)** Effects of Hsp70 (20 µg/ml) on IL-8 secretion into culture medium from HaCaT cells stimulated by IMQ (50 µmol). Control cells were treated with equal amount of PBS. IL-8 levels in the cell culture supernatants were analyzed by ELISA. Results are mean ± SEM of one experiment performed in triplicate. Dot plots overlaid on bar graphs represent individual data points. *P < 0.05, ***P < 0.001.

We further explored direct effects of h-Hsp70 and p-Hsp70 on the proliferation of IMQ-stimulated HaCaT cells. We found that both h-Hsp70 and p-Hsp70 significantly inhibited proliferation of activated keratinocytes in a dose-dependent manner (**Figure 6A**). In addition, h-Hsp70 and p-Hsp70 significantly inhibited secretion of IL-8 from activated keratinocytes (**Figure 6B**).

## Discussion

Major findings of our study included the observation that immunization with Hsp70, particularly the plant-derived form, protected mice from clinical and histological features of IMQ-induced skin inflammation. Here, we found that p-Hsp70 uniquely induced two regulatory T cell populations (CD4^+^CD25^+^/CD4^+^FoxP3^+^) with a concomitant lesser induction of the pro-inflammatory Th17 cell population *in vivo*, as shown by an increased CD4^+^FoxP3^+^:Th17 ratio. However, our observation that p-Hsp70 actually led to inhibition of Th17 cells in anti-CD3/28-stimulated mouse splenocyte cultures argues against a direct effect of p-Hsp70 on Th17 expansion *in vivo* per se. Concurrent induction of Tregs and Th17 cells in Hsp70-immunized mice can potentially be explained by at least two non-exclusive reasons. First, previous studies in psoriatic patients revealed that the former cell type can differentiate into the latter (15). Second, it is also speculated that autologous extracellular Hsp70 plays a dual role in the cellular immune response which may depend on the type of cells that interact with Hsp70 and the type of the disease (4, 16). Our results are consistent with previously published pre-clinical observations. It has been shown that immunization of animals with bacterial Hsp70 and its highly conserved peptides could be regarded as a potential treatment option for autoimmune arthritis *via* induction of Tregs (4, 17–20). Here, the initially *in vivo* observed beneficial effects of Hsp70 vaccination on Tregs and the epidermal turnover, both known to be impaired in patients with psoriasis (8), were also confirmed in *in vitro* experiments. To prove a direct effect of Hsp70 treatment on Treg expansion and reduction in epidermal thickness, anti-CD3/CD28-stimulated spleen cultures and IMQ-treated HaCaT cells were used, respectively. In fact, a direct stimulating action of Hsp70 on regulatory T cells (CD4^+^CD25^+^) and its anti-proliferative effects on HaCaT cells, along with an inhibitory impact on pro-inflammatory and growth-promoting IL-8, were found in these cell culture studies. These results are partly consistent with our previous study showing that intracellular induction of Hsp70 expression by a Hsp90 inhibitor was associated with suppression of IL-8 production in HaCaT cells (21), one of the key cytokines related to psoriasis pathogenesis (22).

This demonstration extends previous reports of a therapeutic potential of targeting Hsp70 by topical approaches in the IMQ mouse model as well as by Hsp70 vaccination in animal models of different autoimmune diseases such as rheumatoid arthritis and lupus erythematosus (4–6, 9, 10). In fact, there is also some evidence from a randomized controlled trial in which intravenous administration of the endoplasmic reticulum Hsp70 family member binding immunoglobulin protein (BiP) to patients with rheumatoid arthritis was associated with clinical and biological improvements in the disease activity (23). It has been suggested that the promoting effects of extracellular Hsp70 on Tregs are driven by major histocompatibility complex (MHC) class II-T cell receptor interactions, toll-like receptor 2 (TLR2) signaling, and PI3K/AKT-JNK-p38 MAPK pathways, but immunomodulation mediated by induction of tolerogenic dendritic cells has also been described (4). Of note, IMQ induces its psoriasis-like phenotype by activating TLR7/8 (11). Thus, it may be hypothesized that Hsp70 not only interacted with Treg-associated TLR2, but also directly interfered as damage-associated molecular pattern with psoriasis-specific signaling of TLR7/8. In this context, it is also worth mentioning that extracellular Hsp70 is known to interact with TLR2/4 on antigen-presenting cells and activate NF-κB (4). However, these previously described pro-inflammatory properties of exogenous Hsp70 may have potentially resulted from the presence of highly immunogenic bacterial endotoxins (e.g., lipopolysaccharides) in recombinant protein preparations produced in bacterial (e.g., *E. coli*) expression systems (4). In contrast, highly pure, substrate- and endotoxin-free m-Hsp70, h-Hsp70, and p-Hsp70 were used in our study.

Hsp70-derived epitopes can also interact with the B cell receptor, leading to production of anti-Hsp70 antibodies (4). Although different anti-Hsp autoantibodies were reported to be elevated in the blood of patients suffering from numerous inflammatory and autoimmune diseases, including rheumatoid arthritis, dermatitis herpetiformis, coeliac disease, and psoriasis, their pathological role and value for prediction of the development of autoimmunity is still obscure (7, 13, 24, 25). In our study, Hsp70 immunization of mice led to a robust humoral anti-Hsp70 response, and anti-Hsp70 antibodies were shown to exert clinical activity associated with decreased pro-inflammatory T cell reposes. Similarly, antibodies toward microbial- and self-Hsp60 were found to be effective in protecting and suppressing arthritis and colitis in rodent models (26, 27). The mechanism by which anti-Hsp70 IgG suppress experimental psoriasis and inhibit polarization of the proinflammatory Th17 population has not been completely solved. We can speculate that anti-Hsp70 antibodies may either expose the disease-modifying epitopes of autologous Hsp70 and/or neutralize those epitopes that are responsible for the induction of Th17 cells.

Lastly, the reason for a higher efficacy of plant-derived Hsp70 over the autologous counterpart in the mouse model of our study requires further elucidation. Nevertheless, we think that it is associated with disease-modifying regulatory CD4^+^CD25^+^ T cells that are expanded only in mice treated with p-Hsp70. Further studies are needed to specify the disease-modifying epitopes of p-Hsp70 for a potential therapeutic purpose.

## Conclusion

We demonstrated that targeting Hsp70 exerts beneficial clinical, immunomodulatory, and anti-proliferative effects in the IMQ mouse model and related *in vitro* assays. These data support further investigations of Hsp70-based treatment modalities in psoriasis and other autoimmune dermatoses.

## Data Availability Statement

The original contributions presented in the study are included in the article. Further inquiries can be directed to the corresponding author.

## Ethics Statement

The animal study and experiments were reviewed and approved by the local authorities of the Animal Care and Use Committee (Bydgoszcz, Poland).

## Author Contributions

ST designed and conceptualized the study. ST, JM, MS, and KB conducted the experiments. ST, JM, MS, KB, and MK analyzed and interpreted the result. ST, KP, ZT, DZ, RL, and MK prepared, revised, and approved the manuscript. All authors contributed to the article and approved the submitted version.

## Funding

This study was supported by the Polish National Science Centre (NCN), grant no. 2017/25/B/NZ6/00305 to ST and the Cluster of Excellence “Precision Medicine in Chronic Inflammation” (EXC 2167) from the Deutsche Forschungsgemeinschaft to DZ and RL.

## Conflict of Interest

The authors declare that the research was conducted in the absence of any commercial or financial relationships that could be construed as a potential conflict of interest.

## References

[1] Saibil H. Chaperone machines for protein folding, unfolding and disaggregation. Nat Rev Mol Cell Biol (2013) 14:630–42. 10.1038/nrm3658 PMC4340576 24026055

[2] Tukaj S Zillikens D Kasperkiewicz M. Heat shock protein 90: a pathophysiological factor and novel treatment target in autoimmune bullous skin diseases. Exp Dermatol (2015) 24:567–71. 10.1111/exd.12760 25980533

[3] Tukaj S Węgrzyn G. Anti-Hsp90 therapy in autoimmune and inflammatory diseases: a review of preclinical studies. Cell Stress Chaperones (2016) 21:213–8. 10.1007/s12192-016-0670-z PMC4786535 26786410

[4] Tukaj S. Heat shock protein 70 as a double agent acting inside and outside the cell: insights into autoimmunity. Int J Mol Sci (2020) 21:E5298. 10.3390/ijms21155298 32722570 PMC7432326

[5] Liu A Ferretti C Shi F-D Cohen IR Quintana FJ La Cava A. DNA vaccination with Hsp70 protects against systemic lupus erythematosus in (NZB×NZW)F1 mice. Arthritis Rheumatol (2020) 72:997–1002. 10.1002/art.41202 31943822

[6] Quintana FJ Carmi P Mor F Cohen IR. Inhibition of adjuvant-induced arthritis by DNA vaccination with the 70-kd or the 90-kd human heat-shock protein: Immune cross-regulation with the 60-kd heat-shock protein. Arthritis Rheumatol (2004) 50:712–20. 10.1002/art.20635 15529360

[7] Wang WM Jin HZ. Heat shock proteins and psoriasis. Eur J Dermatol (2019) 29:121–5. 10.1684/ejd.2019.3526 30998191

[8] Greb JE Goldminz AM Elder JT Lebwohl MG Gladman DD Wu JJ. Psoriasis. Nat Rev Dis Primers (2016) 2:16082. 10.1038/nrdp.2016.82 27883001

[9] Raghuwanshi N Yadav TC Srivastava AK Raj U Varadwaj P Pruthi V. Structure-based drug designing and identification of Woodfordia fruticosa inhibitors targeted against heat shock protein (HSP70-1) as suppressor for Imiquimod-induced psoriasis like skin inflammation in mice model. Mater Sci Eng C Mater Biol Appl (2019) 95:57–71. 10.1016/j.msec.2018.10.061 30573271

[10] Seifarth FG Lax JE-M Harvey J DiCorleto PE Husni ME Chandrasekharan UM. Topical heat shock protein 70 prevents imiquimod-induced psoriasis-like inflammation in mice. Cell Stress Chaperones (2018) 23:1129–35. 10.1007/s12192-018-0895-0 PMC6111098 29616455

[11] van der Fits L Mourits S Voerman JS Kant M Boon L Laman JD. Imiquimod-induced psoriasis-like skin inflammation in mice is mediated via the IL-23/IL-17 axis. J Immunol (2009) 182:5836–45. 10.4049/jimmunol.0802999 19380832

[12] Tukaj S Tukaj Z. Distinct chemical contaminants induce the synthesis of Hsp70 proteins in green microalgae Desmodesmus subspicatus: Heat pretreatment increases cadmium resistance. J Therm Biol (2010) 35:239–44. 10.1016/j.jtherbio.2010.05.007

[13] Tukaj S Görög A Kleszczyński K Zillikens D Kárpáti S Kasperkiewicz M. Autoimmunity to heat shock proteins and vitamin D status in patients with celiac disease without associated dermatitis herpetiformis. J Steroid Biochem Mol Biol (2017) 173:23–7. 10.1016/j.jsbmb.2016.10.002 27760369

[14] Sezin T Zillikens D Sadik CD. Leukotrienes do not modulate the course of Aldara™-induced psoriasiform dermatitis in mice. Acta Derm Venereol (2015) 95:341–2. 10.2340/00015555-1924 24979074

[15] Bovenschen HJ van de Kerkhof PC van Erp PE Woestenenk R Joosten I Koenen HJ. Foxp3+ regulatory T cells of psoriasis patients easily differentiate into IL-17A-producing cells and are found in lesional skin. J Invest Dermatol (2011) 131:1853–60. 10.1038/jid.2011.139 21654831

[16] Tukaj S Mantej J Sobala M Potrykus K Sitko K. Autologous extracellular Hsp70 exerts a dual role in rheumatoid arthritis. Cell Stress Chaperones (2020) 25:1105–10. 10.1007/s12192-020-01114-z PMC7591667 32358783

[17] van Eden W van der Zee R Prakken B. Heat-shock proteins induce T-cell regulation of chronic inflammation. Nat Rev Immunol (2005) 5:318–30. 10.1038/nri1593 15803151

[18] Wieten L Berlo SE Ten Brink CB van Kooten PJ Singh M van der Zee R. IL-10 is critically involved in mycobacterial HSP70 induced suppression of proteoglycan-induced arthritis. PLoS One (2009) 4:e4186. 10.1371/journal.pone.0004186 19142233 PMC2617761

[19] van Herwijnen MJ Wieten L van der Zee R van Kooten PJ Wagenaar-Hilbers JP Hoek A. Regulatory T cells that recognize a ubiquitous stress-inducible self-antigen are long-lived suppressors of autoimmune arthritis. Proc Natl Acad Sci U S A (2012) 109:14134–9. 10.1073/pnas.1206803109 PMC3435203 22891339

[20] Wendling U Paul L van der Zee R Prakken B Singh M van Eden W. A conserved mycobacterial heat shock protein (hsp) 70 sequence prevents adjuvant arthritis upon nasal administration and induces IL-10-producing T cells that cross-react with the mammalian self-hsp70 homologue. J Immunol (2000) 164:2711–7. 10.4049/jimmunol.164.5.2711 10679112

[21] Tukaj S Grüner D Zillikens D Kasperkiewicz M. Hsp90 blockade modulates bullous pemphigoid IgG-induced IL-8 production by keratinocytes. Cell Stress Chaperones (2014) 19:887–94. 10.1007/s12192-014-0513-8 PMC4389849 24796797

[22] Tsai YC Tsai TF. Anti-interleukin and interleukin therapies for psoriasis: current evidence and clinical usefulness. Ther Adv Musculoskelet Dis (2017) 9:277–94. 10.1177/1759720X17735756 PMC5764033 29344110

[23] Kirkham B Chaabo K Hall C Garrood T Mant T Allen E. Safety and patient response as indicated by biomarker changes to binding immunoglobulin protein in the phase I/IIA RAGULA clinical trial in rheumatoid arthritis. Rheumatology (Oxford) (2016) 55:1993–2000. 10.1093/rheumatology/kew287 27498355 PMC5854092

[24] Kasperkiewicz M Tukaj S Gembicki A-J Silló P Görög A Zillikens D. Evidence for a role of autoantibodies to heat shock protein 60, 70, and 90 in patients with dermatitis herpetiformis. Cell Stress Chaperones (2014) 19:837–43. 10.1007/s12192-014-0507-6 PMC4389843 24643797

[25] Mantej J Polasik K Piotrowska E Tukaj S. Autoantibodies to heat shock proteins 60, 70, and 90 in patients with rheumatoid arthritis. Cell Stress Chaperones (2018) 24:283–7. 10.1007/s12192-018-0951-9 PMC6363621 30465159

[26] Ulmansky R Cohen CJ Szafer F Moallem E Fridlender ZG Kashi Y. Resistance to adjuvant arthritis is due to protective antibodies against heat shock protein surface epitopes and the induction of IL-10 secretion. J Immunol (2002) 168:6463–9. 10.4049/jimmunol.168.12.6463 12055266

[27] Ulmansky R Landstein D Moallem E Loeb V Levin A Meyuhas R. A humanized monoclonal antibody against heat shock protein 60 suppresses murine arthritis and colitis and skews the cytokine balance toward an anti-inflammatory response. J Immunol (2015) 194:5103–9. 10.4049/jimmunol.1500023 25904550

